# Risk coefficient model of necroptosis-related lncRNA in predicting the prognosis of patients with lung adenocarcinoma

**DOI:** 10.1038/s41598-022-15189-4

**Published:** 2022-06-29

**Authors:** HuiWei Chen, Zhimin Xie, QingZhu Li, GenYi Qu, NianXi Tan, YuLong Zhang

**Affiliations:** 1 Department of Emergency, Zhuzhou Central Hospital, Zhuzhou, 412007 Hunan China; 2 Department of Stomatology, Zhuzhou Central Hospital, Zhuzhou, 412007 Hunan China; 3 Department of Urology, Zhuzhou Central Hospital, Zhuzhou, 412007 Hunan China; 4 Department of Cardiothoracic Vascular Surgery, Zhuzhou Central Hospital, Zhuzhou, 412007 Hunan China

**Keywords:** Cancer, Oncology, Risk factors

## Abstract

Model algorithms were used in constructing the risk coefficient model of necroptosis-related long non-coding RNA in identifying novel potential biomarkers in the prediction of the sensitivity to chemotherapeutic agents and prognosis of patients with lung adenocarcinoma (LUAD). Clinic and transcriptomic data of LUAD were obtained from The Cancer Genome Atlas. Differently expressed necroptosis-related long non-coding RNAs got identified by performing both the univariate and co-expression Cox regression analyses. Subsequently, the least absolute shrinkage and selection operator technique was adopted in constructing the nrlncRNA model. We made a comparison of the areas under the curve, did the count of the values of Akaike information criterion of 1-year, 2-year, as well as 3-year receiver operating characteristic curves, after which the cut-off value was determined for the construction of an optimal model to be used in identifying high risk and low risk patients. Genes, tumor-infiltrating immune cells, clinical correlation analysis, and chemotherapeutic agents data of both the high-risk and low-risk subgroups were also performed. We identified 26 DEnrlncRNA pairs, which were involved in the Cox regression model constructed. The curve areas under survival periods of 1 year, 2 years, and 3 years of patients with LUAD were 0.834, 0.790, and 0.821, respectively. The cut-off value set was 2.031, which was used in the identification of either the high-risk or low-risk patients. Poor outcomes were observed in patients belonging to the high-risk group. The risk score was the independent predictor of the LUAD outcome (p < 0.001). The expression levels of immune checkpoint and infiltration of specific immune cells were anticipated by the gene risk model. The high-risk group was found to be highly sensitive to docetaxel, erlotinib, cisplatin, and paclitaxel. The model established through nrlncRNA pairs irrespective of the levels of expression could give a prediction on the LUAD patients’ prognosis and assist in identifying the patients who might gain more benefit from chemotherapeutic agents.

## Introduction

One of the major global causes of mortalities that are cancer-related is Lung cancer^1^. In 2020, the number of new lung cancer cases was 2,206,771, while the number of mortalities was 1,796,144^2^. Lung adenocarcinoma (LUAD), a subtype of NSCLC, accounts for ~ 50% of the non-small cell lung cancers (NSCLCs) cases^3,4^. Despite NSCLC molecular-targeting therapy together with chemotherapy having made remarkable advancement, its overall survival rate in the period of 5 years is still lower than 15%^5^. As a result, the identification of robust biomarkers is crucial in predicting the LUAD patients’ prognosis profiles.

Since most cancer is naturally resistant to apoptosis, inducing cell death pathways, e.g., necroptosis, has emerged as a possible therapeutic strategy^6^. Via the mechanism of activating RIPK3 and RIPK1 in the tumor microenvironment (TME), necroptosis, a new programmed type of necrotic cell death distinct from apoptosis, can boost antitumor immunity that is CD8 + leukocyte-mediated^7^. Cancer cells have been found to prefer to avoid necroptosis as a way of their survival. Furthermore, the expression level of low RIPK3 has been shown to be related to an unfavorable prognosis in patients with several cancers^8,9^. From recent research, with regard to the use of necroptosis in cancer control, CD8 + leukocytes, and BATF3 + cDC1 cells were found to be required^7^. These data indicate that necroptosis may act as a possible immunotherapy target for lung adenocarcinoma.

Long non-coding RNAs (lncRNAs), which do not code for proteins, aid in regulating several biological processes, especially in cancers. lncRNAs play a crucial function in human tumors, such as autophagy, tumor initiation, necroptosis, apoptosis, proliferation, cell cycle, and metastasis. lncRNAs can influence gene expression via the mechanism of interfering with protein translation or directly interacting with proteins and other RNA species^10^. lncRNAs have been shown to assist malignancies to avoid immune destruction as well as enhance the inflammation of tumors^11^. lncRNAs contribute to cancer malignant phenotypes through alterations at transcriptomic and genomic levels. They also play a part in changing the immune microenvironment^12^ since lncRNAs control the genes expression that has a relationship with immune cells activation, leading to the infiltration of immune-cell^13^. Moreover, recent evidence suggested that necroptosis-related lncRNAs (nrlncRNAs) can be utilized in predicting the patient prognosis and assist in distinguishing between the cold and hot tumors, thus enhancing the therapy development of Gastric Cancer^14^. Necroptosis-related lncRNAs have not been popularly suggested as a potential therapeutic target to treat lung adenocarcinoma. As a result, more research is needed to thoroughly comprehend the function of necroptosis-related lncRNAs in immunotherapy.

In this study, we used Model algorithms in constructing the risk coefficient model of necroptosis-related long non-coding RNA. Then we identified novel potential biomarkers in the prediction of the sensitivity to chemotherapeutic agents and prognosis of patients with LUAD.

## Materials and methods

### Data acquirement

Transcriptome profiling (RNAseq) data of lung adenocarcinoma and the relevant clinical information were obtained from https://tcga-data.nci.nih.gov/tcga/. It is the website for The Cancer Genome Atlas (TCGA) containing 497 LUAD samples and 54 normal tissue samples adjoining to the tumor. Then, from Ensembl (http://asia.ensembl.org), we acquired the gene transfer format files (GTF) for annotating and differentiating the mRNAs together with lncRNAs from the transcriptome data.

### Identifying necroptosis-related lncRNAs

The necroptosis-related gene set M24779.gmt was obtained from http://www.gseamsigdb.org/gsea/index.jsp, the website for Gene Set Enrichment Analysis (GSEA). Subsequently, it was added to the genes that are necroptosis-related from the prior reports. Using the Pearson correlation analysis and co-expression strategy, lncRNAs with a co-expression correlation coefficient > 0.4 and p-value < 0.001 were defined as nrlncRNAs. We utilized the “limma” R package for the purpose of performing analysis of the differential expression of the acquired nrlncRNAs. The nrlncRNAs showing a log fold change (FC) > 1.0 along with false discovery rate (FDR) < 0.05 were identified as differentially expressed nrlncRNAs (DEnrlncRNAs).

### Construction of DEnrlncRNA pairs

DEnrlncRNAs were cyclically singly paired, and the parameter value was defined as α value of 0 or 1. The lncRNA pair value was 1 in the case where the lncRNA A expression was more than that of a sample of lncRNA B; else, it was 0. Then, the created 0-or-1 matrix was subjected to additional screening.

### Risk model establishment for risk score assessment

For the purpose of screening lncRNAs related to patient survival from the nrlncRNA pool (p < 0.05), a univariate Cox proportional hazard regression analysis was performed. Then, for 1000 cycles, the least absolute shrinkage and selection operator (LASSO) regression analysis was conducted through the use of a p-value < 0.05 together with cross-validation of 10-folds. To prevent overfitting, stimulation was set up 1000 times for each cycle in a random manner. For the purpose of conducting Cox proportional hazard regression analysis, we chose pairs that contained a frequency of more than 100 times, and the best lncRNA pairs were chosen for the construction of the Cox risk coefficient model. By risk score calculation, we computed the AUC value of every model and drew the 1-, 2-, and 3-year receiver operating characteristics (ROC) curves that are dependent on time of the model.

$$ {\text{Risk Score}} = \sum\limits_{i = 1}^{n} {{\text{Risk}}\,\,{\text{coefficient}}\,\,i \times {\text{nrlncRNA}}\,\,{\text{Expression}}\,\,i.} $$

Groups of low and high-risk were constructed based on the optimal fitting of the Akaike information criterion (AIC).

### Constructed risk model validation

The Kaplan–Meier analysis was conducted in investigating the survival differences between patients in both groups. With the aid of the “survival” and “survminer” R packages, the survival curve was drawn for visualization. The Chi-square test was performed in finding out the relationship between the model and clinical factors. We then carried out the Wilcoxon rank-sum test for assessing the associations between several subgroups of clinical indicators and the risk score. To obtain a clear comprehension of the data, we utilized “limma” and “ggpubr” in R packages. To validate that the model can be utilized on LUAD patients as a clinical prognostic predictor that is independent, Cox multivariate and univariate regression analyses were executed on clinical correlation features as well as the risk score. To envisage this data, the R “survival” package was put into use. For the purpose of comparing the accuracy of the risk score and clinically relevant features in predicting survival profiles and outcomes, we made a comparison of the ROC curves acquired from a follow-up lasting 1 year with ROC curves of indicators that are clinically relevant in the same chart.

### Tumor-Infiltrating immune cells correlation analysis

Current techniques were utilized in calculating the status of the immune infiltration in the TCGA samples, including XCELL (http://xCell.ucsf.edu/)^15^, QUANTISEQ (http://icbi.at/quantiseq)^16^, TIMER (version 2.0; http://timer.cistrome.org/)^17^, MCPCOUNTER, CIBERSORT (http://cibersort.stanford.edu/)^18^, EPIC (http://epic.gfellerlab.org)^19^, and CIBERSORT-ABS to perform analysis of the correlation between infiltration of immune cells and the risk score. We assessed the differences existing between the high-risk and low-risk groups in their tumor-infiltrating immune cell content by the Wilcoxon signed-rank test. Furthermore, we did an analysis on the spearman correlation to find out the relationship between the risk score and the infiltration levels of the immune cells. The “limma”, “scales,” “ggplot2,” as well as “ggtext” packages in R were utilized in data visualization.

### Immunosuppressive molecules expression analysis related with ICIs

The “limma” and “ggpubr” packages in R were utilized to find out if there were substantial differences in gene expression that are ICI-related between the two groups. After this, visualization of data was performed.

### Chemotherapeutic agents correlation analysis

The conventional chemotherapeutic drugs, including erlotinib, cisplatin, paclitaxel, docetaxel, gefitinib, individually or in combination, were selected in determining if there existed a difference in chemotherapeutic agents response based on LUAD patients belonging to both groups. We utilized the drug’s half-inhibition rate (IC50) as an index in measuring the sensitivity to the drug. The “limma,” “ggpubr,” “ggplot,” and “pRRophetic” packages in R were used in data analyses and visualization. This study utilized R software (version 4.0.0: http://www.r-project.org) for conducting statistical analyses, and Supplementary Table S7 provides the specific functions of the R package.

### Ethics approval and consent to participate

Not applicable, data was collected from public data repositories.

### Guidelines statement

All experimental protocols were performed in accordance with the relevant guidelines and regulations and adhered to the Declaration of Helsinki.

## Results

### Necroptosis-related lncRNAs of patients with LUAD

**Figure 1:**
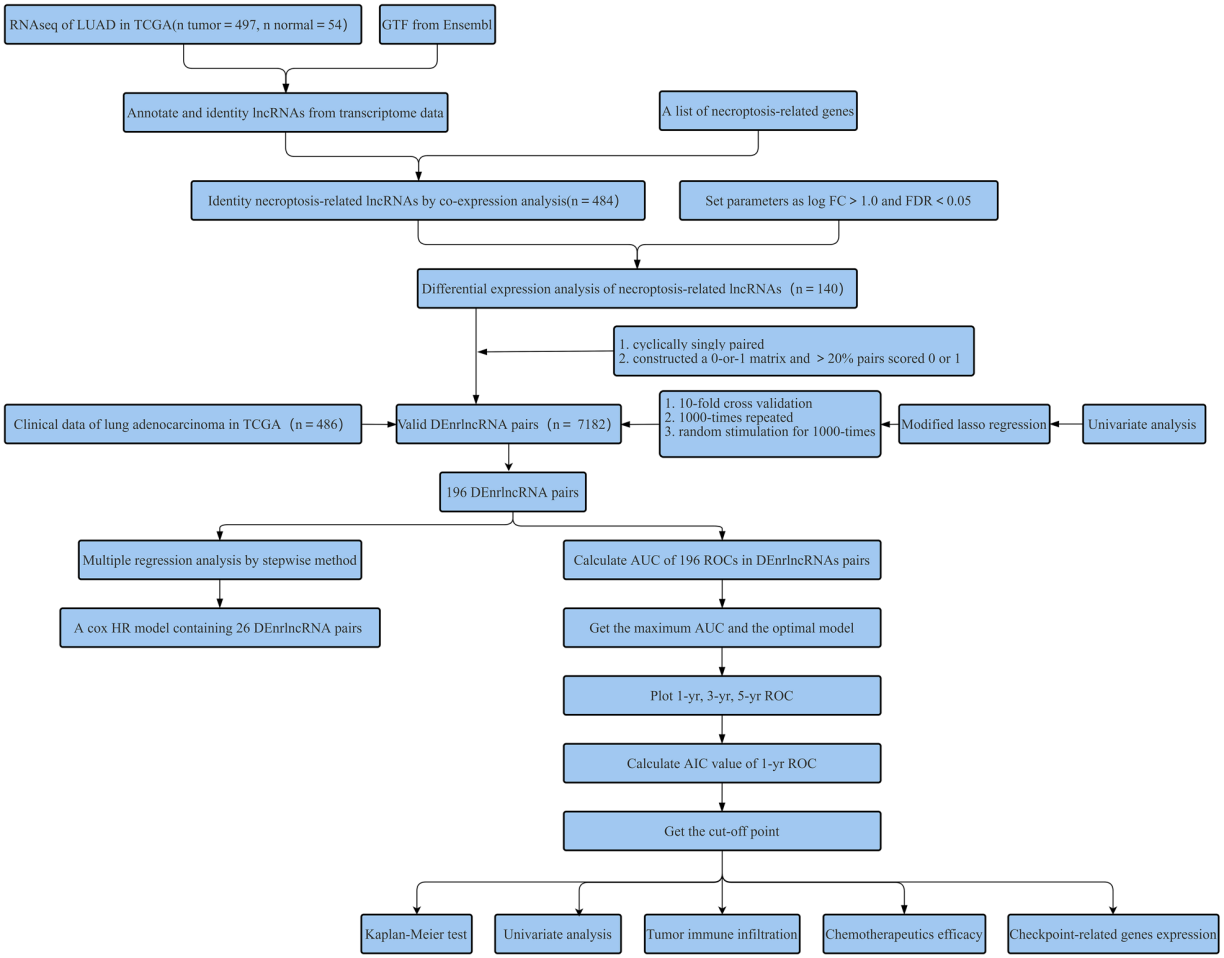
The study’s flow chart.

**Figure 2:**
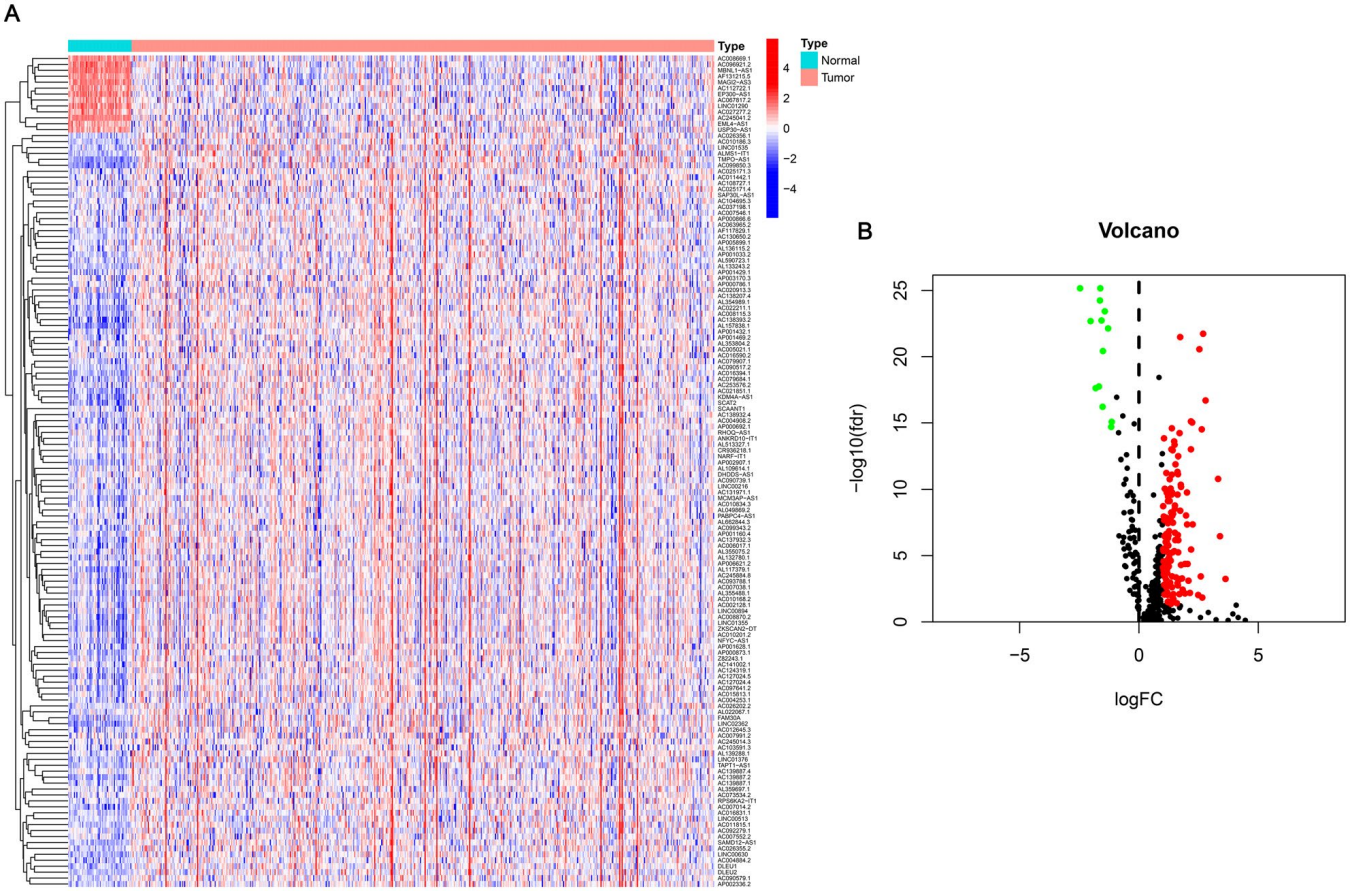
Heat map and differential expression analysis of necroptosis-related lncRNAs in LUAD. (**A**) Heat map of Necroptosis-related lncRNA genes of normal tissues and LUAD tissues. Upregulation is shown by Red, while blue downregulation is shown by Blue. (**B**) Necroptosis-related lncRNAs in LUAD together with normal tissue are shown as a volcano plot. Red dots: upregulated with significant differential expression; green dots: downregulated with significant differential expression; black dots: no significant difference.

As shown in the flow chart (Fig. 1), the TCGA database was used in retrieving the LUAD transcriptome data. Data that contained no duplicates and follow-up time were not included in the present research, and 497 LUAD samples and 54 normal tissue samples adjacent to the tumor were considered. From GSEA and previous reports, we acquired a 67 necroptosis-related genes (nr-genes) profile (Supplementary Table S1). Co-expression analysis was conducted between lncRNAs and nr-genes that were known, and differentially expressed lncRNAs (|LogFC|> 1.0 and p < 0.05) between cancer and normal samples were identified. There was a total of 484 identified nrlncRNAs (Supplementary Table S2), and 140 obtained DEnrlncRNAs; of the 140, there were 127 upregulated and 13 downregulated DEnrlncRNAs (Fig. 2A,B, and Supplementary Table S3).

### Establishment of differentially expressed necroptosis-related lncRNA pairs and the risk coefficient model

**Figure 3:**
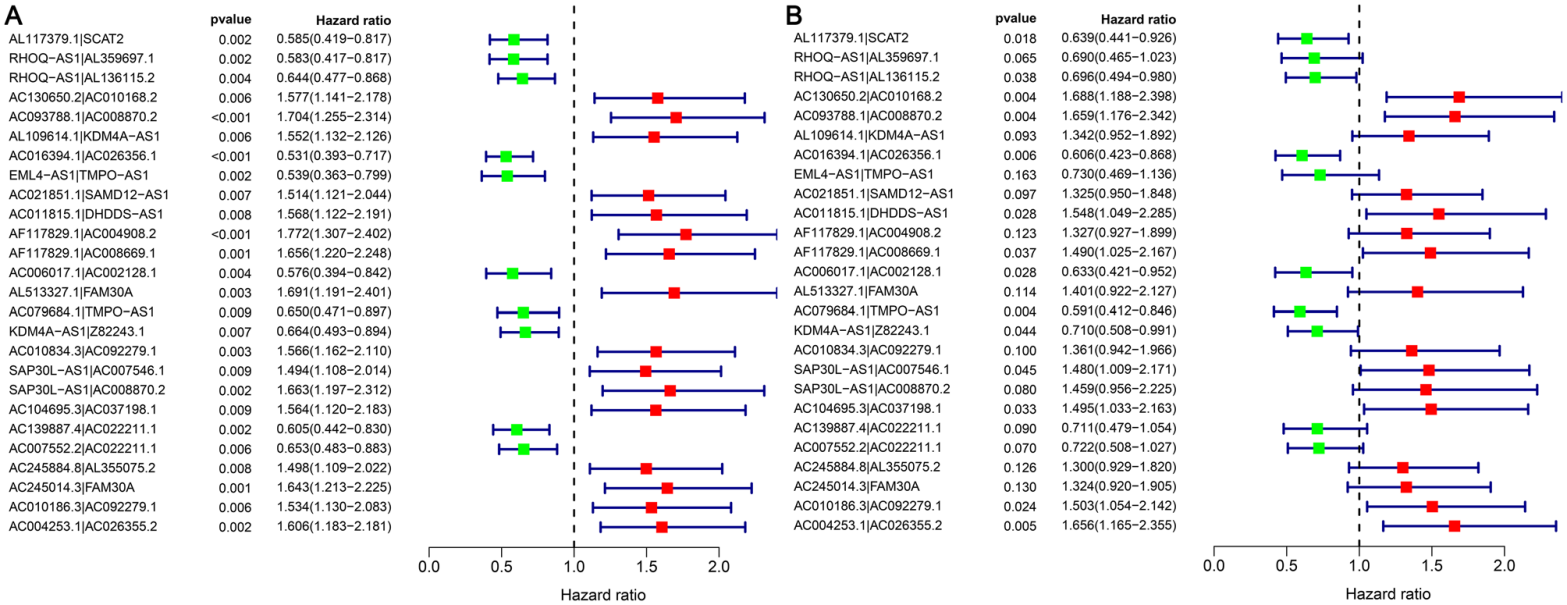
Results obtained from Cox regression analysis based on 26 necroptosis-related lncRNA pairs associated with the outcome of LUAD. (**A**) Forest plot of the 26 necroptosis-related lncRNA pairs associated with the outcome of LUAD by performing Cox univariate regression analysis. (**B**) Forest plot of Cox multivariate regression analysis of 26 necroptosis-related lncRNA pairs associated with LUAD. Risk factors are highlighted in red, while protective factors are highlighted in green.

By making the 140 DEnrlncRNAs match from several cycles, 7182 differentially expressed necroptosis lncRNA pairs, in total, were acquired (Supplementary Table S4). By performing univariate Cox (uni-Cox) regression analysis, we identified 196 DEnrlncRNA pairs that could significantly affect LUAD patient survival (Supplementary Table S5). Next, 26 DEnrlncRNAs pairs were identified for the risk coefficient model building by performing the LASSO regression analysis. These 26 DEnrlncRNA pairs were then analyzed by performing multivariate as well as univariate Cox regression analysis (Fig. 3A,B) to obtain the risk factor for each necroptosis-related lncRNA pair (Table 1).

**Table 1:** 26 pairs of prognostic necroptosis-related lncRNA pairs multivariate COX regression analysis results.

LncRNAs	Coefficient	HR	HR.95L	HR.95H	P-value
AL117379.1|SCAT2	− 0.4473	0.6393	0.4414	0.9258	0.0179
RHOQ-AS1|AL359697.1	− 0.3710	0.6900	0.4655	1.0228	0.0647
RHOQ-AS1|AL136115.2	− 0.3629	0.6957	0.4940	0.9796	0.0377
AC130650.2|AC010168.2	0.5233	1.6876	1.1877	2.3978	0.0035
AC093788.1|AC008870.2	0.5064	1.6593	1.1756	2.3420	0.0040
AL109614.1|KDM4A-AS1	0.2945	1.3424	0.9523	1.8923	0.0927
AC016394.1|AC026356.1	− 0.5008	0.6060	0.4229	0.8684	0.0064
EML4-AS1|TMPO-AS1	− 0.3142	0.7302	0.4693	1.1363	0.1635
AC021851.1|SAMD12-AS1	0.2816	1.3252	0.9502	1.8481	0.0971
AC011815.1|DHDDS-AS1	0.4371	1.5482	1.0490	2.2849	0.0277
AF117829.1|AC004908.2	0.2826	1.3266	0.9266	1.8991	0.1227
AF117829.1|AC008669.1	0.3991	1.4905	1.0251	2.1671	0.0366
AC006017.1|AC002128.1	− 0.4567	0.6334	0.4214	0.9520	0.0281
AL513327.1|FAM30A	0.3370	1.4007	0.9224	2.1270	0.1138
AC079684.1|TMPO-AS1	− 0.5265	0.5907	0.4124	0.8460	0.0041
KDM4A-AS1|Z82243.1	− 0.3426	0.7099	0.5085	0.9911	0.0442
AC010834.3|AC092279.1	0.3084	1.3612	0.9423	1.9662	0.1003
SAP30L-AS1|AC007546.1	0.3922	1.4802	1.0092	2.1707	0.0447
SAP30L-AS1|AC008870.2	0.3776	1.4588	0.9563	2.2253	0.0797
AC104695.3|AC037198.1	0.4019	1.4946	1.0327	2.1632	0.0331
AC139887.4|AC022211.1	− 0.3417	0.7105	0.4789	1.0543	0.0896
AC007552.2|AC022211.1	− 0.3256	0.7223	0.5079	1.0270	0.0701
AC245884.8|AL355075.2	0.2626	1.3003	0.9290	1.8200	0.1258
AC245014.3|FAM30A	0.2807	1.3241	0.9203	1.9050	0.1304
AC010186.3|AC092279.1	0.4071	1.5025	1.0541	2.1417	0.0244

*HR* hazard ratio, *HR.95L* 95% CI lower limit, *HR.95H* 95% CI upper limit.

### Assessment of the prognostic predictive performance of the risk model

**Figure 4:**
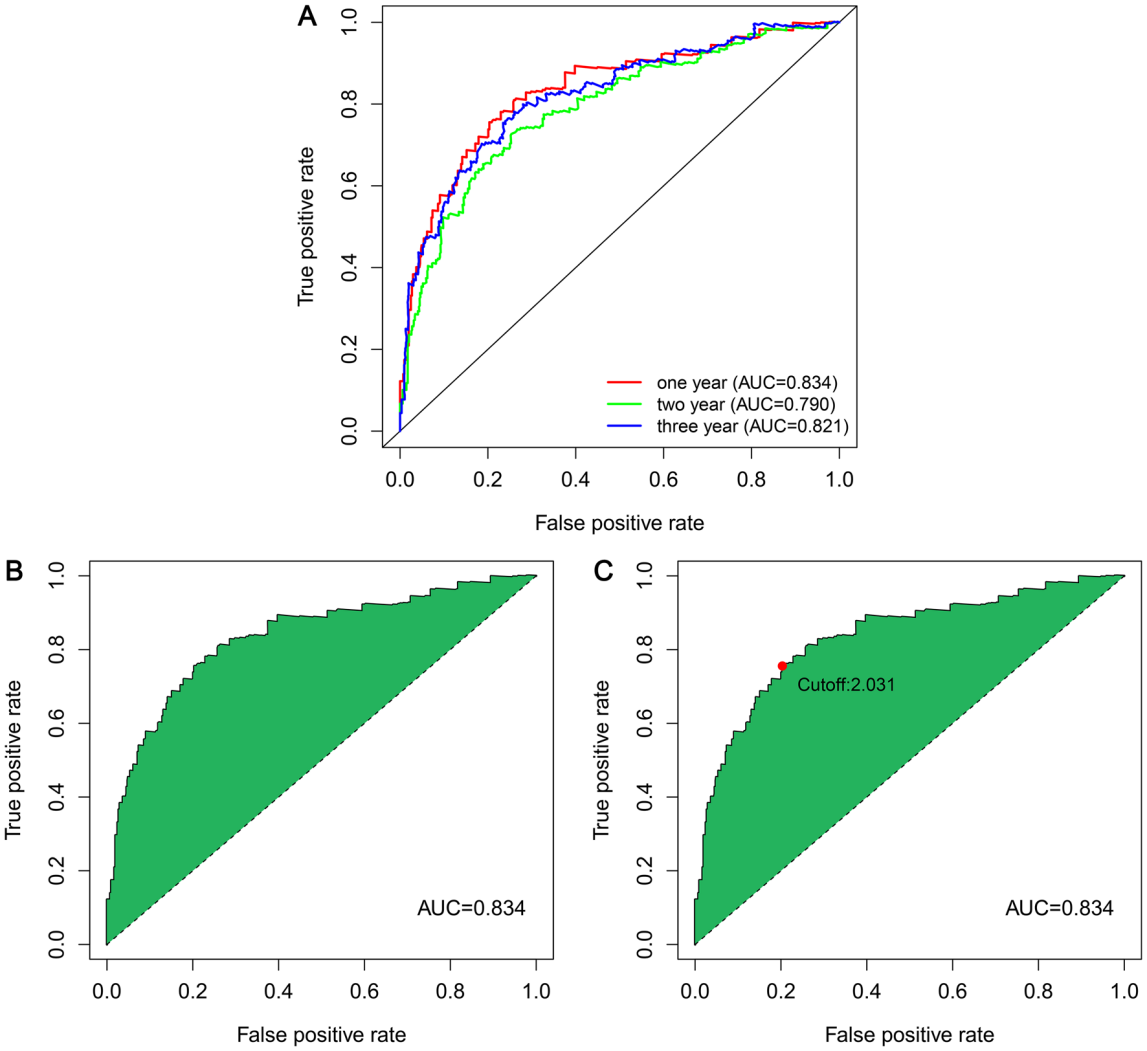
The curves of ROC were obtained after the determination of the risk coefficient model based on necroptosis-related lncRNA pairs of LUAD. (**A**) The model was used in obtaining ROC curves during the periods of 1 year, 2 years, as well as 3 years. The values for AUC were greater than 0.790. (**B**) A ROC curve of one-year containing the highest value of AUC was established with the aid of the model. (**C**) 2.031 was the cut-off value, which differentiates between high- and low-risk patients using the best fit.

The 26 prognostic DEnrlncRNA pairs that were selected were utilized in constructing patients’ receiver operator characteristic (ROC) curves during the periods of 1, 2, and 3 years (Fig. 4A). The areas under the curve (AUC) during the periods of 1, 2, and 3 years were found to be 0.834, 0.790, and 0.821, respectively, which also contained a predictive significance. The 1-year area under the curve (AUC) was calculated of 0.834, which was the largest AUC (Fig. 4B). The cut-off value was computed based on the best fit and found to be 2.031(Fig. 4C). This cut-off value was used in differentiating between high- and low-risk groups of patients with LUAD. Accordingly, the low-risk group contained 338 patients who participated in the group, whereas the high-risk group contained 126 patients.

### Correlation analysis of clinical features with the aid of the risk model

**Figure 5:**
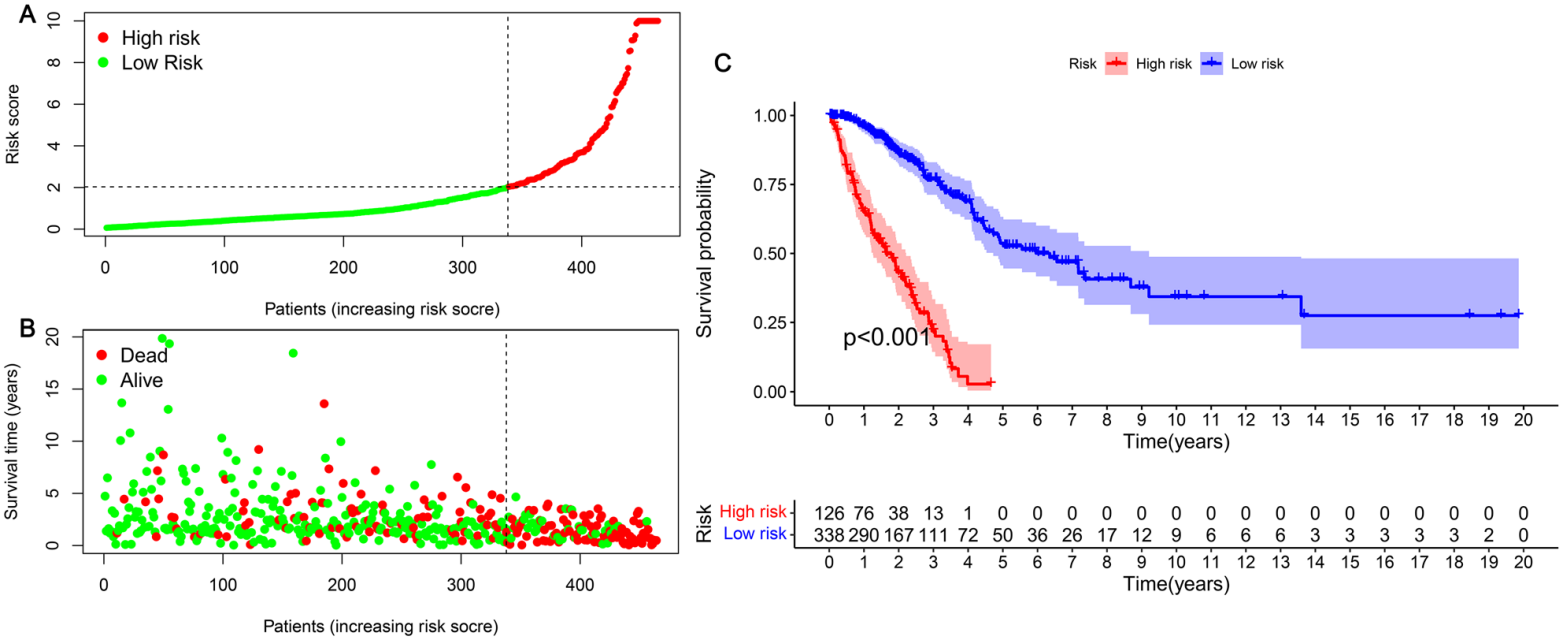
Risk coefficient model for the prognosis anticipation of LUAD. (**A**) To classify patients into low- and high-risk groups, the risk score was utilized. (**B**) Scatter plot of risk score and survival outcome for each patient. (**C**) Construction of the Kaplan–Meier curve on the basis of the survival status of patients belonging to both groups.

R was used in analyzing the correlation between risk-subgroup patients and risk scores (Fig. 5A). We obtained the relationship of the patients on their risk coefficient score as well as the survival status (Fig. 5B). Subsequently, through the use of the survival status from both groups, the construction of the Kaplan–Meier curve was achieved (Fig. 5C). The findings indicated that the patients' rate of survival in the low-risk group was substantially greater in comparison to that of the group of high-risk (p < 0.001). Moreover, patients belonging to the low-risk group presented a survival time that was significantly longer in comparison to that in the high-risk group (p < 0.001).

**Figure 6:**
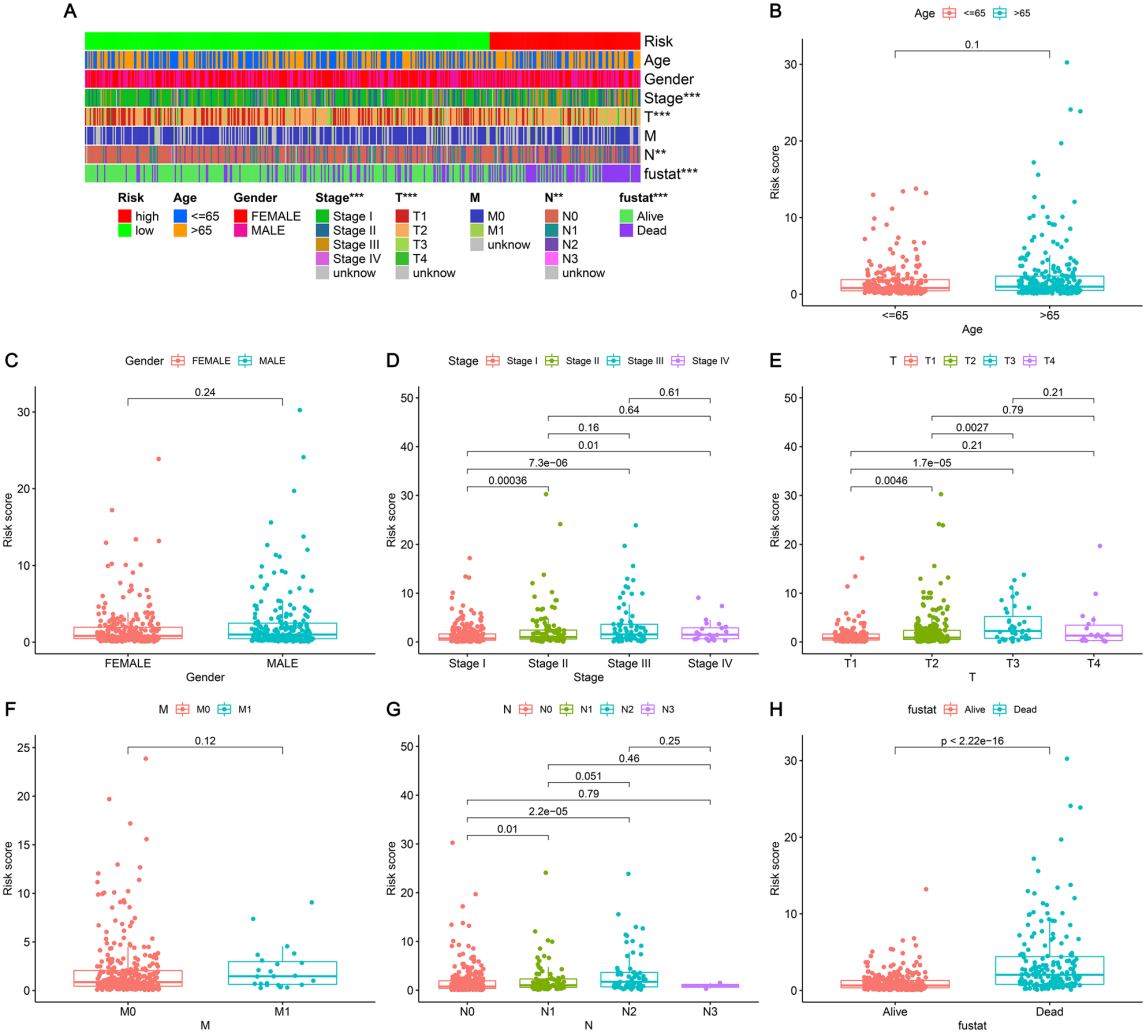
Risk coefficient model of LUAD for clinical correlation analysis. (**A**) The clinical correlation heatmap, (**B**) age, (**C**) gender, (**D**) clinical-stage, (**E**) T stage, (**F**) M stage, (**G**) N stage, and (**H**) survival status.

For the purpose of examining the correlation between clinical features and LUAD risk, we conducted multiple chi-square tests. The Wilcoxon signed-rank test assisted us in obtaining a heatmap (Fig. 6A). From the findings, it was evident that there was a significant correlation between the risk coefficient score and LUAD patients’ survival status (p < 0.001), T stage (p < 0.001), N stage (p < 0.01), and tumor stage (p < 0.001). The scatter diagrams of clinical characteristics indicated that risk scores were significantly different by clinical-stage (Fig. 6D), T stage (Fig. 6E), N stage (Fig. 6G), and survival status (Fig. 6H), while Age (Fig. 6B), Gender (Fig. 6C), and M stage (Fig. 6F) were not significantly different.

**Figure 7:**
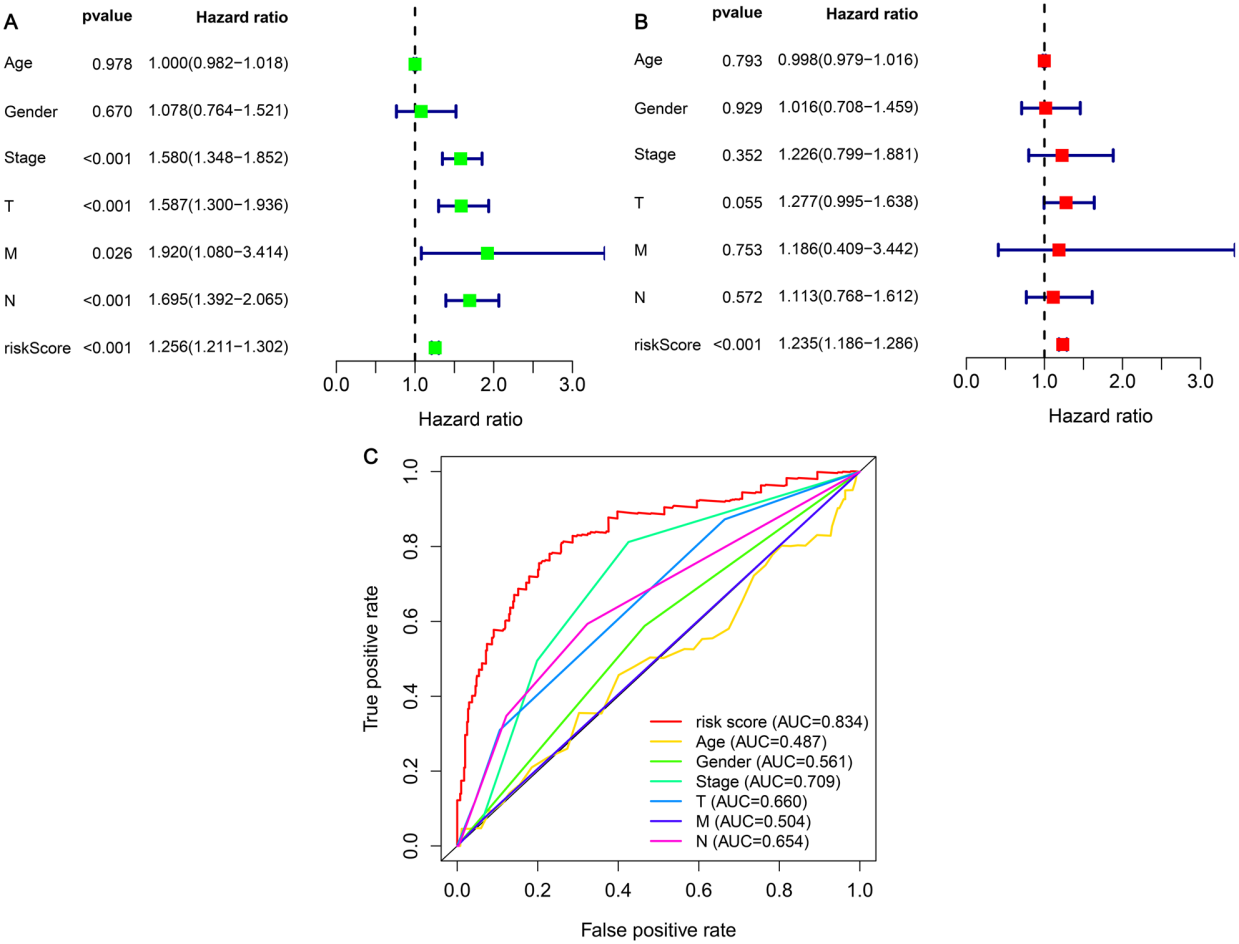
Cox regression analysis of clinical correlation characteristics and integrated ROC curves. (**A**) Clinical characteristics of Cox univariate regression analysis indicated that Stage, T stage, M stage, N stage, and risk score were correlated with the outcome of LUAD. (**B**) Cox multivariate analysis showed that risk scores were predictors of outcome in an independent manner. (**C**) The comparison of risk coefficient score and clinical characteristics showed that Stage (AUC = 0.709) and risk coefficient score (AUC = 0.834) had the highest predictive efficacy.

From the findings of the study, it was evident that the clinical-stage of LUAD patients (p < 0.001, HR = 1.580, 95% CI [1.348–1.852]), T stage (p < 0.001, HR = 1.587, 95% CI [1.300–1.936]), M stage (p = 0.026, HR = 1.920, 95% CI [1.080–3.414]), N stage (p < 0.001, HR = 1.695, 95% CI [1.392–2.056]), and risk score (p < 0.001, HR = 1.256, 95% CI [1.211–1.302]) indicated that differences were significant according to the results of the univariate Cox regression analysis (Fig. 7A). However, the risk score (p < 0.001, HR = 1.235, 95% CI [1.186–1.286]) was the only factor whose presentation was a prognostic predictor that was independent by performing the multivariate Cox regression analysis (Fig. 7B). We did a comparison on the ROC curve of clinical features and the risk coefficient score during the period of 1 year (Fig. 7C). The result showed that the patients’ risk score (AUC = 0.834) and stage (AUC = 0.709) had the highest predictive efficacy.

### Immune-cell infiltration and risk coefficient model correlation

**Figure 8:**
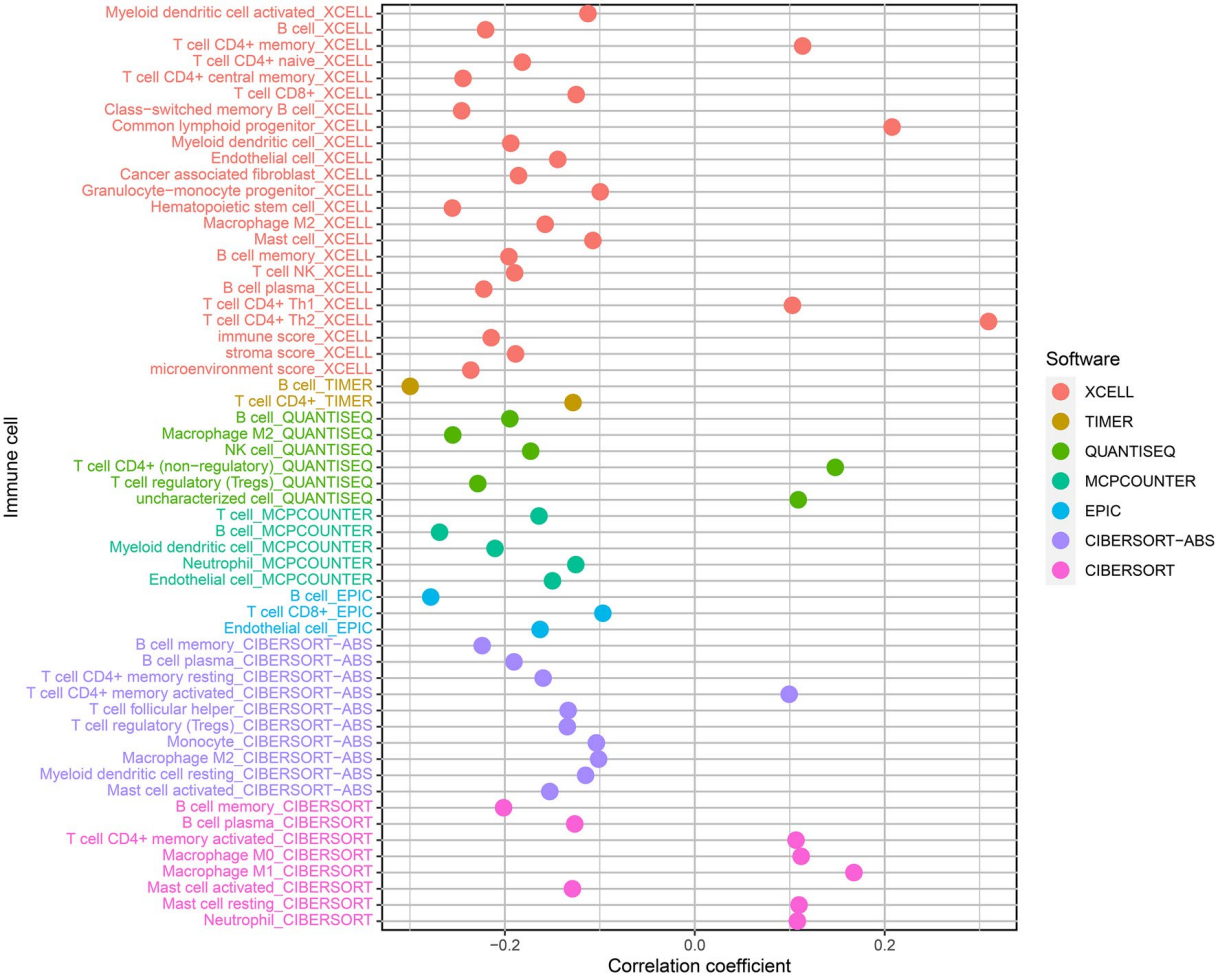
Correlations between immune cell infiltrations and risk score of LUAD samples.

We assessed if there was a relationship between the tumor immune microenvironment and the model (Fig. 8). The findings are recorded in Supplementary Table S6. By performing Spearman correlation analysis, we established a positive correlation existed on the tumor-infiltrating immune cells when compared to the high-risk group, including common lymphoid progenitors, resting mast cells, CD4 + T cells, macrophage M1, uncharacterized cells, macrophage M0, as well as neutrophils (Supplementary Fig. S1).

### Correlation between risk coefficient model and genes

**Figure 9:**
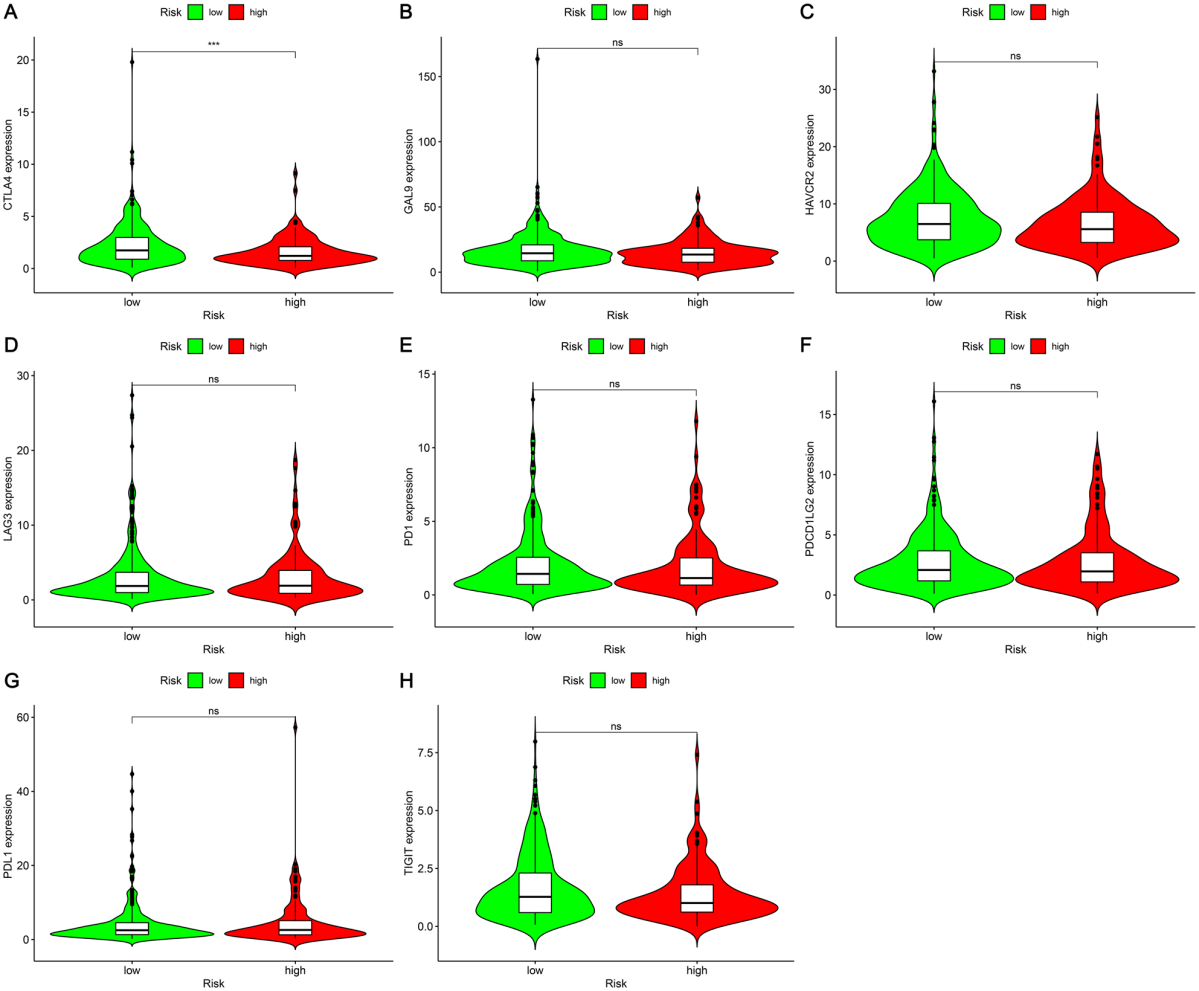
The relationship between LUAD patients’ genes and risk model. The levels of expression of (**A**) CTLA4; (**B**) GAL9; (**C**) HAVCR2; (**D**) LAG3; (**E**) PD1; (**F**) PDCD1LG2; (**G**) PDL1; (**H**) TIGIT in high-risk and low-risk LUAD subjects. *Ns* not significant; ***p < 0.001.

We also did an analysis of the correlation of genes to that of the risk coefficient model and found out that a relationship between high-risk scores and the expression level of CTLA4 existed (p < 0.001, Fig. 9A). Nevertheless, GAL9 (p > 0.05, Fig. 9B), HAVCR2 (p > 0.05, Fig. 9C), LAG3 (p > 0.05, Fig. 9D), PD1 (p > 0.05, Fig. 9E), PDCD1LG2 (p > 0.05, Fig. 9F), PDL1 (p > 0.05, Fig. 9G), and TIGIT (p > 0.05, Fig. 9H) indicated that the correlation was not significant.

### The correlation between the risk coefficient model and chemotherapeutic agents

**Figure 10:**
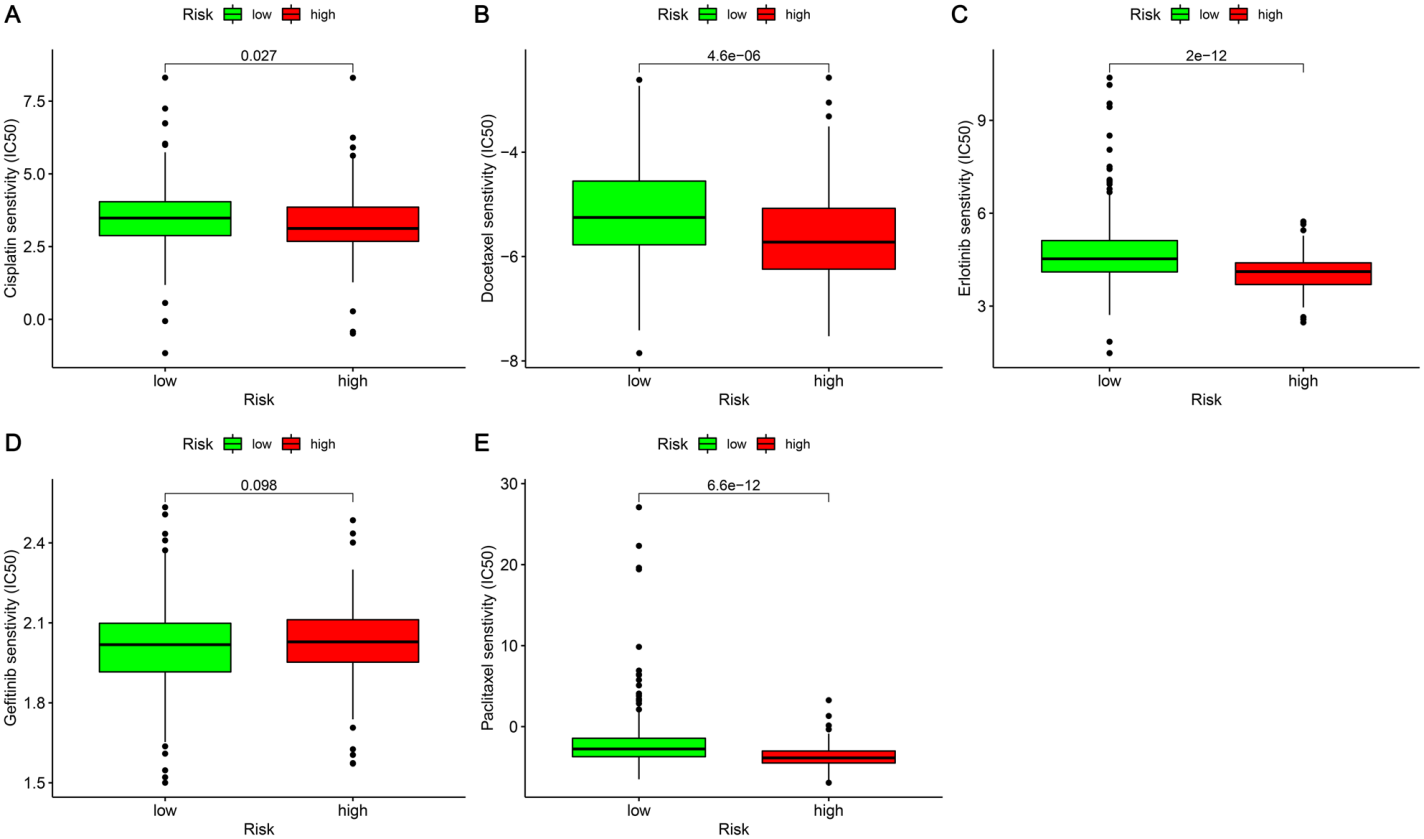
The relationship between LUAD patients’ chemotherapeutic agents and risk model. In comparison to the low-risk group, the high-risk group was correlated to a higher sensitivity to (**A**) Cisplatin; (**B**) Docetaxel; (**C**) Erlotinib; (**E**) Paclitaxel. However, (**D**) Gefitinib was not significantly different between the two groups.

Moreover, we investigated the correlation between the risk coefficient model and the sensitivity to chemotherapeutic agents. To assess the drugs’ efficacy, we utilized IC50. Lower IC50, indicating that the sensitivity was higher. According to these findings, we established that there was a correlation between the high-risk group and the higher sensitivity to cisplatin (Fig. 10A), docetaxel (Fig. 10B), erlotinib (Fig. 10C), and paclitaxel (Fig. 10E). There was no significant difference in the sensitivity to gefitinib in both groups (Fig. 10D).

## Discussion

Recent research reports have set up signatures based on lncRNAs for the purpose of evaluating the prognosis of patients with cancer. lncRNA-related models of LUAD, such as immune-related lncRNAs^20–22^, autophagy-related lncRNAs^23–25^, pyroptosis-related lncRNAs^26^, and methylation-driven lncRNAs^27^, were reported in previous studies. In this study, we constructed the models of risk coefficient, which were essential in the assessment of the LUAD patients’ prognosis based on the nrlncRNA pairs.

In the present research, we firstly obtained LUAD patients’ nr-gene and lncRNAs data from GSEA and TCGA, did an analysis of the differential co-expression in establishing the DEnrlncRNAs, and then performed lncRNA pairs validation through cyclically single pairing them together with a matrix of 0-or-1. Second, we acquired each sample’s risk coefficient of patients with LUAD and then constructed a risk coefficient model by performing multivariate regression, LASSO regression analyses, as well as Cox multivariate and univariate regression analyses. Third, we computed each ROC’s AUC value in obtaining the optimal model fit and then obtained the critical value based on optimal fitting of the Akaike information criterion (AIC), which was utilized in identifying the difference existing in the high and low-risk groups. The novel model had the clinical practicability benefit in differentiating between the cases belonging to both groups.

We performed the correlation analyses in assessing the efficacy and accuracy of the constructed risk coefficient model, including tumor-infiltrating immune cells, survival, genes, clinical characteristics, and chemotherapeutic agents, and found that the model algorithm worked well.

To find out the correlation between the risk scores and tumor-infiltrating immune cells, we utilized seven techniques that are generally accepted to estimate the immune infiltrating cells, including TIMER, XCELL, CIBER-SORT, QUANTISEQ, EPIC, MCPCOUNTER, as well as CIBERSORT-ABS, and found out that the relationship between the tumor-infiltrating immune cells, such as CD4 + T cells, resting mast cells, common lymphoid progenitors, uncharacterized cells, macrophage M0, macrophage M1, and neutrophils and the high-risk group was positive (p < 0.01). Wu et al*.*^28^ proposed that *LINC00665* played a critical role in enhancing the infiltration levels of macrophages, dendritic cells, and inhibited regulatory T cells to avoid exhaustion. T-cell. Xu et al*.*^29^ established that HK3 enhanced the infiltration of macrophages and monocytes that presented the antigens of the cell surface and regulated the debilitating T cells’ critical genes (*PD1* and *CTLA4*), thus having an effect on the process of immune escape.

In cancer immunotherapy, it was found that necroptosis participated highly in the immunity of the antitumor. Good performance was evident from the prognostic signature that was necroptosis-related on the basis of four genes (*EZH2*, *TLR4*, *TRAF2*, and *PGAM5*) in the prediction of the prognosis of patients with stomach adenocarcinoma^30^. Immune checkpoint inhibitors (ICIs) include anti-cytotoxic T lymphocyte-associated antigen 4 (CTLA-4) and anti-programmed cell death protein 1/programmed cell death ligand 1 (PD-1/PD-L1). Recent studies have identified novel immune checkpoint targets such as lymphocyte activation gene 3 (LAG-3), T cell immunoglobulin and ITIM domain (TIGIT), T cell immunoglobulin and mucin-containing domain 3 (TIM-3), hepatitis A virus cellular receptor 2 (HAVCR2) gene and the TIM-3 ligand galectin-9 (Gal-9), etc. Cytotoxic T lymphocyte-associated protein 4 (CTLA-4) expressed by T cells is recognized as a key immune checkpoint for autoimmunity and cancer therapeutic targets. CTLA-4 is a member of the immunoglobulin-associated receptor family, which Suppresses T cell activation and responsible for all aspects of T cell immune regulation. The generation of specific monoclonal antibodies illustrates the controlling role of CTLA-4 in T cell responses. CTLA-4 can mediate negative regulation of T cell activation by competing with the co-stimulatory receptor CD28 for binding to its co-ligands B7.1 and B7.2. It can also be regulated by promoting Treg development and function.After activation, CTLA-4 expression is induced on CD4 + Foxp3− (forkhead box P3) and CD8 + Foxp3− conventional T cells, while CTLA-4 constitutively expressed by CD4 + Foxp3 + Treg cells^16^. We believe that there are differences between various immune cells and immune-related phenotypes. According to the findings of the present research, we observed the expression levels of CTLA4 were elevated in samples from patients belonging to the high-risk group, which can be utilized as a potential therapeutic target.

Tumor microenvironment changes may be linked to the development of immune-targeted drug resistance, making it crucial to discover sensitive drugs for clinical therapy. The correlation analysis of chemotherapeutic agents showed that the sensitivity to cisplatin, docetaxel, erlotinib, and paclitaxel was greater in the group of high-risk in comparison to that of the group of low-risk. It was shown that necroptosis induction in the immune checkpoint blockade (ICB) and tumor microenvironment may have a synergistic impact on enhancing a long-term tumor rejection^7^. The phosphorylation or lack of caspase 8 was essential for Paclitaxel-triggered necroptosis in lung adenocarcinoma cells. When epithelial-mesenchymal transition (EMT) was triggered, a novel lncRNA, called lncCRLA, was markedly upregulated, which inhibited RIPK1-induced necroptosis by interfering with the RIPK1-RIPK3 interaction by binding to the RIPK intermediate domain^31^.

Necroptosis is a form of regulated cell death regulated by RIP1, RIP3, and MLKL. Inducing necroptosis in mice with orthotopic pancreatic cancer increased the survival time and attenuated tumor growth, stroma, and metastasis^32^. lncRNAs have been established to affect tumor cell growth from several previous research, which is crucial for clinical therapy as well as patient prognosis^33^. For example, via the mechanism of inhibiting miR-150-5p, lncRNA *LINC00673* regulates the invasion, epithelial-mesenchymal transition, migration, as well as the proliferation of non-small cell lung cancer^34^, while *LINC00472* inhibited EMT via binding to YBX1 and affecting the cell’s mechanical features, and as a result, obstructing its invading and metastasizing ability^35^. The lncRNA *MIF-AS1* enhanced the proliferation of tumor cells while reducing apoptosis in digestive system cancer^36^. Experiments have shown that the lncRNA that is necrosis-related has been shown from several experiments to target miR-873; moreover, RIPK1/RIPK3 plays a role in the regulation of the cardiomyocyte necroptosis^37^. Another study found that when HCC expresses lncRNA *LINC00176*, miRNAs, such as *miR-9* and *miR-185*, are produced and downregulate the mRNAs they target, which enhances the necroptosis of liver cancer cells^38^.

The novel insights about nrlncRNAs could assist us in gaining a clear understanding of the LUAD mechanism, which could have a crucial role in the treatment. However, in the present research, there were some disadvantages together with limitations. For initial analysis, there was insufficient raw data and thus more clinical data are needed. The sample size was small. The risk factor model lacked external data validation, which reduced the reliability of the model, so further validation needs to be performed.

Finally, the present research showed that nrlncRNAs and the risk coefficient might be utilized in predicting the prognosis of patients with LUAD and assist in identifying those patients who might benefit from chemotherapeutic agents.

## Supplementary Information

Supplementary Figure 1.

Supplementary Table 1.

Supplementary Table 2.

Supplementary Table 3.

Supplementary Table 4.

Supplementary Table 5.

Supplementary Table 6.

Supplementary Table 7.

Supplementary Legends.

## Data Availability

The datasets analysed during the current study are available in the [The Cancer Genome Atlas (TCGA)] repository, [persistent web link to datasets] [https://portal.gdc.cancer.gov/repository].

## Abbreviations

TCGA: The Cancer Genome Atlas
DEnrlncRNAs: Differently expressed necroptosis-related long noncoding RNAs
NSCLC: Non-small cell lung cancer
AUC: Area under the curve
ICIs: Immune checkpoint inhibitors
LUAD: Lung adenocarcinoma
nr-genes: Necroptosis related genes
FDR: False discovery rate
IC50: Half-inhibition rate
AIC: Akaike information criterion
lncRNA: Long noncoding RNA
ICB: Immune checkpoint blockade
EMT: Epithelial-mesenchymal transition
RNAseq: Transcriptome profiling
ROC: Receiver operating characteristics
LASSO: Least absolute shrinkage and selection operator
TME: Tumor microenvironment
uni-Cox: Univariate Cox
HCC: Hepatic cell carcinoma
CI: Confidence interval
GTF: Gene transfer format
GSEA: Gene set enrichment analysis
FC: Fold change
nrlncRNAs: Necroptosis-related long non-coding RNAs
HR: Hazard ratio
