# Supplementary figures and images for: Risk coefficient model of necroptosis-related lncRNA in predicting the prognosis of patients with lung adenocarcinoma
Source: Sci Rep. 2022 Jun 29;12:11005. doi: 10.1038/s41598-022-15189-4 (PMC9243036; doi:10.1038/s41598-022-15189-4)

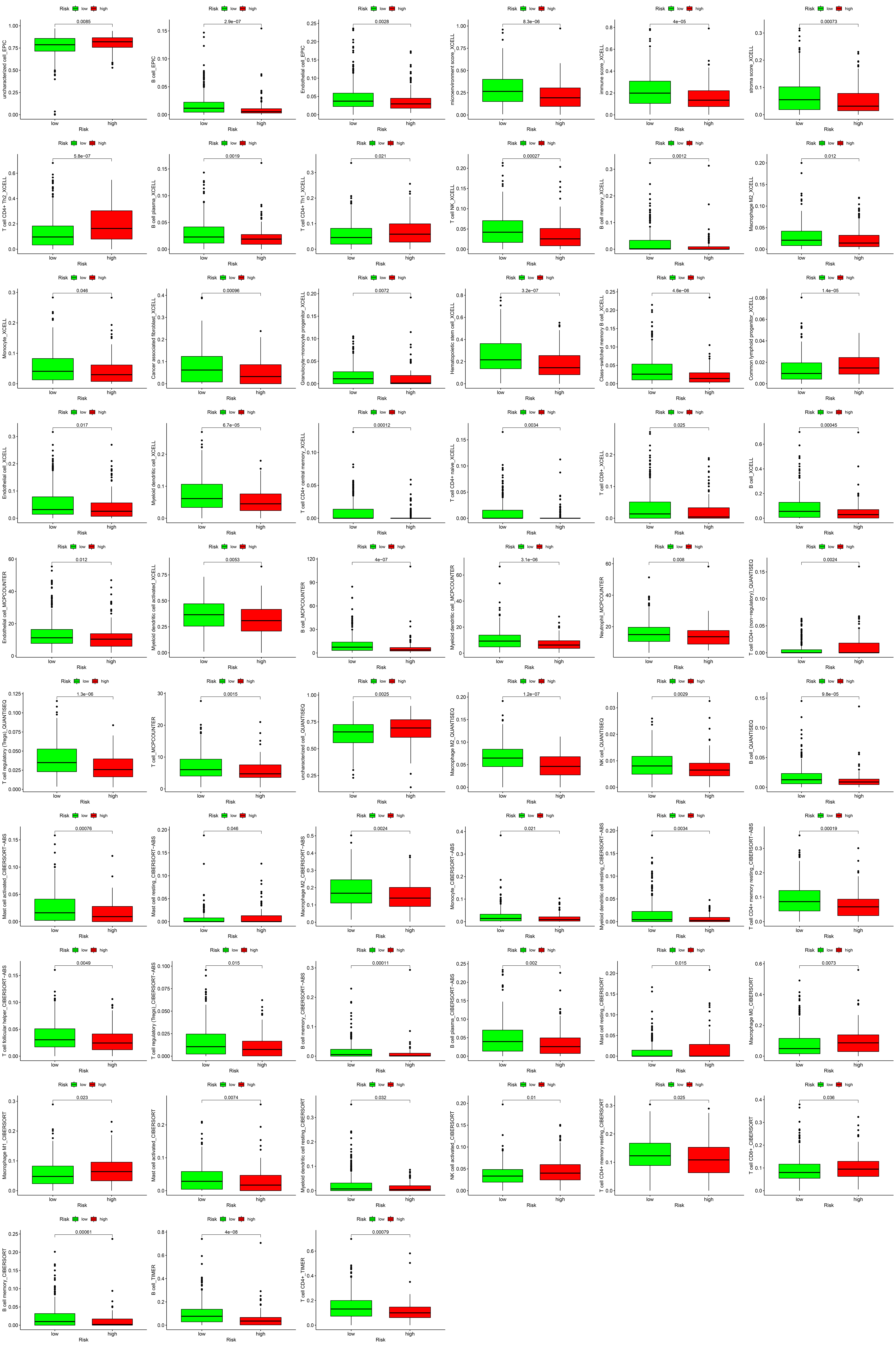

Supplement: Supplementary file 1 — Supplementary Figure 1. [file 41598_2022_15189_MOESM1_ESM.tif]
