# Supplementary material for: Risk coefficient model of necroptosis-related lncRNA in predicting the prognosis of patients with lung adenocarcinoma
Source: Sci Rep. 2022 Jun 29;12:11005. doi: 10.1038/s41598-022-15189-4 (PMC9243036; doi:10.1038/s41598-022-15189-4)
Supplement: Supplementary file 8 — Supplementary Table 7. [file 41598_2022_15189_MOESM8_ESM.docx]

The specific functionalities of the R packages

| R packages | Function |
| --- | --- |
| “survival” packages | Data set that contains the survival time (in months), survival status and other information of patients. |
| “survminer” packages | The survminer R package provides functions for facilitating survival analysis and visualization. |
| “limma” packages | limma is a library for the analysis of gene expression microarray data, especially the use of linear models for analysing designed experiments and the assessment of differential expression. limma provides the ability to analyse comparisons between many RNA targets simultaneously in arbitrary complicated designed experiments. Empirical Bayesian methods are used to provide stable results even when the number of arrays is small. The linear model and differential expression functions apply to all gene expression technologies, including microarrays, RNA-seq and quantitative PCR. |
| “ggpubr” packages | ggplot2, by Hadley Wickham, is an excellent and flexible package for elegant data visualization in R. However the default generated plots requires some formatting before we can send them for publication.  The 'ggpubr' package provides some easy-to-use functions for creating and customizing 'ggplot2'- based publication ready plots. |
| “scales” packages | One of the most difficult parts of any graphics package is scaling, converting from data values to perceptual properties. The inverse of scaling, making guides (legends and axes) that can be used to read the graph, is often even harder! The idea of the scales package is to implement scales in a way that is graphics system agnostic, so that everyone can benefit by pooling knowledge and resources about this tricky topic. |
| “ggplot2” packages | A system for 'declaratively' creating graphics, based on "The Grammar of Graphics". You provide the data, tell 'ggplot2' how to map variables to aesthetics, what graphical primitives to use, and it takes care of the details. |
| “ggtext” packages | The ggtext package implements both geoms (geom_richtext(), geom_textbox()) and theme elements (element_markdown(), element_textbox()) for improved text rendering with ggplot2. |
| “ggplot” packages | ggplot() initializes a ggplot object. It can be used to declare the input data frame for a graphic and to specify the set of plot aesthetics intended to be common throughout all subsequent layers unless specifically overridden. |
| “pRRophetic” packages | an R package for prediction of clinical chemotherapeutic response from tumor gene expression levels |
