# Supplementary material for: Risk coefficient model of necroptosis-related lncRNA in predicting the prognosis of patients with lung adenocarcinoma
Source: Sci Rep. 2022 Jun 29;12:11005. doi: 10.1038/s41598-022-15189-4 (PMC9243036; doi:10.1038/s41598-022-15189-4)
Supplement: Supplementary file 9 — Supplementary Legends. [file 41598_2022_15189_MOESM9_ESM.docx]

**Additional files:**

Supplementary table 1. Necroptosis-related genes list.

Supplementary table 2. Necroptosis-related lncRNAs list.

Supplementary table 3. Dysregulated necroptosis-related lncRNA pairs in LUAD.

Supplementary table 4. A total of 7182 differentially expressed necroptosis lncRNA pairs.

Supplementary Table 5. Necroptosis-related lncRNA pairs that substantially affected the prognosis of patients with LUAD, according to the results of the univariate cox regression analysis.

Supplementary Table 6. The correlation between tumor-infiltrating immune cells and the risk sore.

Supplementary table 7. The specific functionalities of the R packages.

Supplementary figure 1. Immune-cell infiltration in high- and low-risk LUDA samples.
